# Population Risk Predictors of Major Adverse Kidney Events in Patients with Focal Segmental Glomerulosclerosis from the CURE-CKD Registry

**DOI:** 10.21203/rs.3.rs-5844460/v1

**Published:** 2025-05-06

**Authors:** Susanne B. Nicholas, Lindsey M. Kornowske, Cami R. Jones, Kenn B. Daratha, Radica Z. Alicic, Christina L. Reynolds, Joshua J. Neumiller, Mark E. Bensink, Wu Gong, Keith C. Norris, Katherine R. Tuttle

**Affiliations:** University of California, Los Angeles; Providence Inland Northwest Health; Providence Inland Northwest Health; Providence Inland Northwest Health; Providence Inland Northwest Health; Providence Inland Northwest Health; Washington State University; Travere Therapeutics Inc; Travere Therapeutics Inc; University of California, Los Angeles; Providence Inland Northwest Health

**Keywords:** Focal segmental glomerulosclerosis, Population level predictors, kidney function, Health insurance, Health care utilization, Clinical outcomes, Clinical decision-making

## Abstract

**Background:**

Predictors of major adverse kidney events (MAKE) in focal segmental glomerulosclerosis (FSGS) have not been previously explored within large, real-world populations. The study aim was to evaluate population-level predictors of MAKE for patients with FSGS from health system data.

**Methods:**

The study population was derived from electronic health records from Providence and University of California Los Angeles Health. Cox proportional hazards models were used to estimate the effects of clinical and non-clinical variables including age, gender, race and ethnicity, health system, health insurance, healthcare utilization, estimated glomerular filtration rate (eGFR), diabetes, hypertension, and prescription medications as predictors of MAKE defined as: ≥40% eGFR decline, kidney failure (eGFR <15 mL/min/1.73 m^2^, administrative codes for kidney failure, dialysis, or transplant) and death.

**Results:**

Adults with FSGS (N=629) were 54% (n=342) men and 53±17 (mean±SD) years old. Baseline eGFR was 60±30 mL/min/1.73 m^2^, while median (interquartile range) urine albumin/creatinine ratio (UACR) and urine protein/creatinine ratio (UPCR) were 1,430 (520–2,630) mg/g and 1.6 (0.5–3.9) g/g, respectively. Angiotensin converting enzyme inhibitors or angiotensin receptor blockers were prescribed to 76% (n=475), while corticosteroids and other immunomodulators were prescribed in 47% (n=297) and 12% (n=74), respectively. MAKE were observed in 42% (n=262) of study participants over a median of 2.9 (1.4–4.5) years. Higher hazard for MAKE was associated with above-median UACR or UPCR (HR [95% CI] (3.46 [2.28–5.23]) in patients with available measures, prescription for non-corticosteroid immunomodulator (1.87 [1.32–2.65]), non-commercial health insurance (1.78 [1.36–2.33]), hospitalization (1.64 [1.25–2.15]), lower eGFR per 10 mL/min/1.73 m^2^ 1.25 [1.18–1.32]), number of outpatient visits (1.03 [1.01–1.05]) and lower hazard for MAKE was associated with older age (0.89 [0.82–0.98]).

**Conclusions:**

Substantial loss of kidney function or kidney failure occurred in more than four in ten patients with FSGS by a median of three years. MAKE were predicted by unique population level factors, such as healthcare utilization and insurance type, which may help to identify patients with FSGS, who could most benefit from diagnostic testing and interventions to improve clinical outcomes.

## Background

Focal segmental glomerulosclerosis (FSGS) is the most common cause of nephrotic syndrome and the most common primary glomerular disease in patients with kidney failure in the United States (US) [1]. FSGS describes a set of heterogeneous glomerular diseases in which some, but not all, glomeruli become sclerosed (focal), and involved glomeruli are only partially affected (segmental) [2, 3]. As FSGS progresses, the entire glomerulus becomes affected with more diffuse lesions and extensive accumulation of hyalinosis, podocyte effacement, and vascular obstruction [2, 4]. Although the exact incidence of FSGS is unknown, significant increases in kidney biopsy proven FSGS have ranged from 17% per five years between 1994 to 2003, to as high as 41% per five years between 2004 to 2013 [5]. More males than females are affected with FSGS [6].

FSGS may present with varying degrees of proteinuria and different rates of progression leading to major adverse kidney events (MAKE). Due to disease heterogeneity, identification of patient characteristics that predict progression and therapeutic response has been challenging. The mainstay of therapy for FSGS has traditionally included angiotensin converting enzyme (ACE) inhibitors or angiotensin receptor blockers (ARBs), glucocorticoids, and calcineurin inhibitors.[7] However, novel therapies have opened doors for various treatment options such that knowledge of key risk predictors would be instrumental in stratifying patients for timely interventions. Although several studies have previously examined characteristics associated with FSGS progression, these studies have been limited to small sample sizes and have reported varying results [8–12]. For instance, the presence of nephrotic syndrome predicts response to immunosuppression, and the histopathologic presence of interstitial inflammation was found to be a risk factor for kidney failure in a cohort of active-duty members of the US Department of Defense [13]. Nevertheless, specific predictors of MAKE have not been explored at a population level. The study aim was to evaluate clinical and non-clinical predictors of MAKE defined by ≥ 40% decline in estimated glomerular filtration rate (eGFR), kidney failure, and death in a large real-world population with FSGS.

## Methods

### Study Population

**Figure 1:**
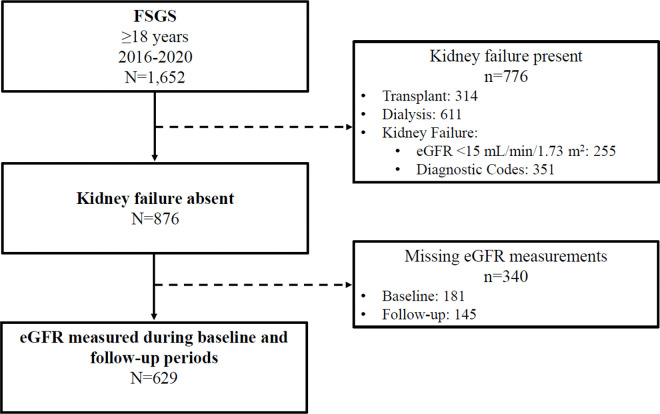
STROBE diagram for FSGS cohort selection. FSGS-focal segmental glomerulosclerosis, eGFR-estimated glomerular filtration rate

The Center for Kidney Disease Research, Education, and Hope (CURE-CKD) Registry [14, 15] contains demographics, physical and laboratory measures, prescription medications, and administrative codes from the Providence and UCLA electronic health records (EHRs) for patients with chronic kidney disease (CKD) and at-risk for CKD by virtue of having diabetes, prediabetes, or hypertension. Identification of FSGS was based on International Classification of Diseases (ICD) 9/ICD 10 diagnostic codes. Baseline characteristics were determined from data for the 12-months prior to FSGS identification (Additional file 1) between January 1, 2016, and December 31, 2022. Patients were excluded for kidney failure or kidney replacement therapy defined by baseline eGFR < 15 mL/min/1.73 m^2^ or a diagnosis or procedure code indicative of kidney failure, transplant, or dialysis (Additional file 2). At least one eGFR measure (Chronic Kidney Disease Epidemiology 2021 equation) [16] during the baseline and follow-up periods were required for study inclusion (N = 629, Fig. 1). Diabetes and hypertension were identified as previously described for CURE-CKD [14]. All-cause death was ascertained from Providence EHR data using the Social Security Death Index [17], and from UCLA EHR data using the California death index [18]. Race and ethnic identity were as reported in the EHR, and medication use was defined by an active prescription at baseline. Time-to-event analysis followed study participants from the index time until the first MAKE, or last encounter through December 31, 2022.

### Statistical analysis

Categorical variables are reported as frequencies and percentages. Continuous normally distributed variables are reported as mean ± standard deviation (SD), while continuous skewed variables are reported as median and interquartile range (IQR; Table 1). Median MAKE survival was estimated using the Kaplan-Meier method. Separate Kaplan-Meier estimates were constructed for the outcomes of all-cause death, ≥ 40% eGFR decline, kidney failure (eGFR < 15 mL/min/1.73 m^2^, administrative codes for kidney failure, dialysis, kidney transplant), and for composite MAKE grouped by eGFR category.

Cox proportional hazards models were used to estimate the effects of clinical and non-clinical variables on the hazard of MAKE in patients with FSGS. Unadjusted and adjusted models were constructed from a set of baseline clinical variables including eGFR (per – 10 mL/min/1.73 m^2^), diabetes and hypertension status (yes/no), and prescription medications including ACE inhibitors, ARBs, corticosteroids, and other immunomodulators (yes/no; including biologics, calcineurin inhibitors, cytotoxic agents, mammalian target of rapamycin inhibitors, corticotropin agents, and pyrimidine synthesis inhibitors). Demographic variables included age (per 10 years), gender (women or men), and race and ethnicity (non-White versus White, non-Hispanic). Health care variables were health system (Providence versus UCLA Health), primary payer for health insurance (non-commercial versus commercial), hospitalization (yes/no) and frequency of outpatient visits (number) in the 12-month baseline period. Exploratory subgroup analyses included 1) a fully adjusted Cox model including status above versus below the cohort median urine albumin/creatinine ratio (UACR; 1,430 mg/g) or urine protein/creatinine ratio (UPCR; 1.6 g/g) for the subset of participants with baseline period UACR or UPCR measurements (N = 299) and 2) unadjusted Cox models to examine association of variables with MAKE by eGFR category. Alpha was selected as < 0.05 *a priori* to define statistical significance. A Bonferroni correction adjusted for multiple comparisons used to test an interaction term for each variable and eGFR category (P < 0.001) in the exploratory subgroup analysis. Analyses were performed in R version 4.2.2 [19] with the Survival package version 3.5–5 [20] and Prodlim package version 2023.03.3 [21].

## Results

### Baseline Characteristics

The FSGS cohort (N = 629) was 54% men (n = 342/629) and 53 ± 17 years of age (Fig. 1, Table 1). Race identity was Black in 8.7% (n = 55/629), Asian in 13.2% (n = 83/629) and White in 52% (n = 327/629). Ethnic identity was Hispanic or Latino(a) in 4.5% (n = 28/629). The proportion of participants with commercial health insurance as the primary payer was 59% (n = 371/629), while 27% (n = 169/629) of patients had Medicare and 12% (n = 75) had Medicaid [22]. Hypertension and diabetes were present in 85% (n = 535/629) and 39% (n = 245/629), respectively. Baseline eGFR (mean ± SD) was 60 ± 30 mL/min/1.73 m^2^ and the median (IQR) UACR and UPCR were 1,430 (520–2,630) mg/g and 1.6 (0.5–3.9) g/g, respectively. Prescription medications included ACE inhibitors/ARBs (76%, n = 475/629), corticosteroids (47%, n = 297/629), and other immunomodulators (12%, n = 74/629; Table 1). The characteristics of patients identified with FSGS according to eGFR category at baseline for model variables are shown in Additional file 3.

### Rates and Predictors of Major Adverse Kidney Events

**Figure 2:**
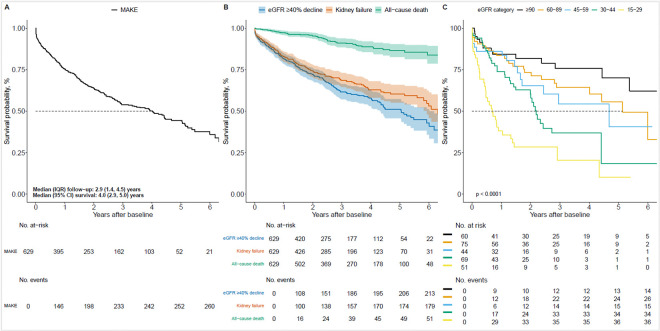
Survival probability of MAKE in patients with FSGS Kaplan-Meier survival curves. **A.** Primary composite MAKE outcome ≥40% eGFR decline, eGFR <15 mL/min/1.73 m^2^, dialysis or transplant, and death in patients with FSGS (N=629). **B.** Individual components for the primary composite MAKE outcome. **C**. Subgroup analysis according to eGFR category. MAKE-major adverse kidney events, eGFR-estimated glomerular filtration rate

MAKE were observed for 42% (n/N = 262/629) of study participants over a median (IQR) follow-up of 2.9 (1.4–4.5) years (Fig. 2A). Median (95% confidence interval [CI]) survival without MAKE was 4.0 (2.9–5.0) years. First MAKE was 51% (133/262) for ≥ 40% eGFR decline, 28% (74/262) for eGFR < 15 mL/min/1.73 m^2^, 11% (28/262) for dialysis, 5% (13/262) for kidney transplant, and 5% (14/262) for all-cause death (Table 2). The frequency of individual MAKE components by eGFR category showed that 40% eGFR decline events were more common among groups with higher eGFR categories (≥ 90 – 45 mL/min/1.73 m^2^), and that kidney failure events, dialysis and all-cause death were proportionately greater among those in the lower eGFR categories (30–44 and 15–29 mL/min/1.73 m^2^; Additional file 4).

Kaplan-Meier estimates for deaths (8%; n/N = 52/629), kidney failure (29%; n/N = 181/629), and ≥ 40% eGFR decline (34%; n/N = 215/629) as independent outcomes are shown in Fig. 2B. The results of Kaplan-Meier estimates of MAKE survival by eGFR category indicate that groups with lower eGFR categories (30–44 and 15–29 mL/min/1.73 m^2^) tended to have lower survival compared to those with higher eGFR (≥ 90, 60–89 and 45–59 mL/min/1.73 m^2^; Fig. 2C.

**Figure 3:**
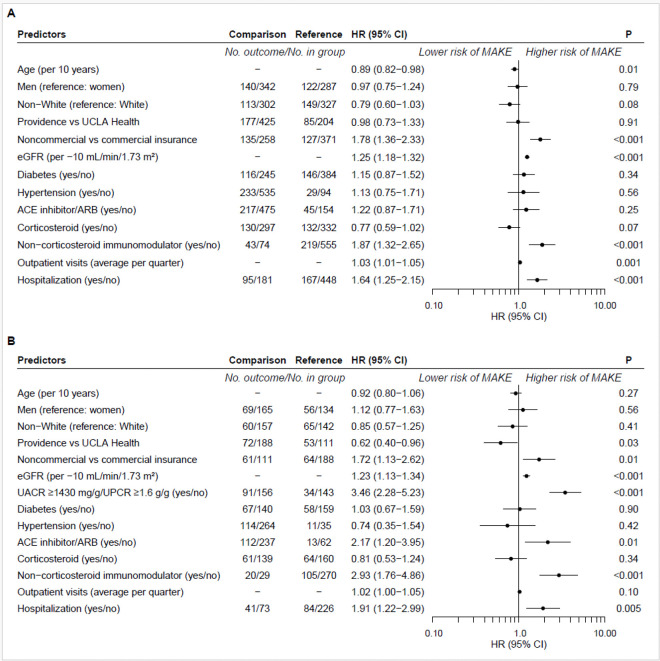
Predictors of MAKE in patients with FSGS. Adjusted Cox proportional hazards models **A.**Main model with full cohort. **B.** Secondary model including subset with baseline UACR or UPCR measurements. MAKE-major adverse kidney events, HR-hazard ratio, CI-confidence interval, UCLA-University of California Los Angeles, eGFR-estimated glomerular filtration rate, ACE-angiotensin converting enzyme, ARB-angiotensin II receptor blocker, UACR-urine albumin/creatinine ratio, UPCR-urine protein/creatinine ratio (in A and B use non-corticosteroid immunomodulator)

In the main analysis, baseline prescription for non-commercial health insurance (HR = 1.78, 95% CI = 1.36–2.33), lower baseline eGFR per 10 mL/min/1.73 m^2^ (HR = 1.25, 95% CI = 1.18–1.32), non-corticosteroid immunomodulators (hazard ratio [HR] = 1.87, 95% CI = 1.32–2.65), more frequent outpatient visits (HR = 1.03, 95% CI = 1.01–1.05) and hospitalization (HR = 1.64, 95% CI = 1.25–2.15) predicted greater hazard for MAKE (Fig. 3A). Notably, older age was associated with lower hazard for MAKE (HR = 0.89, 95% CI = 0.82–0.98).

In the exploratory analysis of the subgroup with UACR or UPCR measurements during the baseline period, similar overall relationships as with the main analysis were observed (Additional file 2). UACR ≥ 1,430 mg/g or UPCR ≥ 1.6 g/g predicted higher MAKE hazard (HR = 3.46, 95% CI = 2.28–5.23; Fig. 3B). Other predictors were prescriptions for non-corticosteroid immunomodulators (HR = 2.93, 95% CI = 1.76–4.86) and ACE inhibitors or ARBs (HR = 2.17, 95% CI = 1.20–3.95), hospitalization (HR = 1.91, 95% CI = 1.22–2.99), non-commercial health insurance (HR = 1.72, 95% CI = 1.13–2.62), and lower eGFR per 10 mL/min/1.73 m^2^ (HR = 1.23, 95% CI = 1.13–1.34). Differing from the main analysis, patients from Providence compared with UCLA Health had lower hazard for MAKE (HR = 0.62, 95% CI = 0.40–0.96).

The exploratory analysis of unadjusted Cox proportional hazards models by eGFR category showed that variable associations with MAKE were generally consistent with the main analysis (Additional file 5). There was no evidence for interaction by eGFR category after correction for multiple comparisons.

## Discussion

Our study assessed unique clinical and non-clinical population-level predictors of MAKE, not previously known to impact clinical outcomes or facilitate risk stratification in patients with FSGS. We showed that MAKE were highly common among a diverse cohort of adult patients with FSGS in contemporary clinical practice settings at two large health systems in the US. MAKE occurred in 42% of patients with FSGS and a mean baseline eGFR of 60 mL/min/1.73 m^2^ over a relatively short median follow-up of 2.9 years. Of patients who experienced MAKE, 51% had a ≥ 40% decline in eGFR, while nearly as many (44%) had kidney failure defined by eGFR < 15 mL/min/1.73 m^2^, dialysis, or transplant as a first event. Higher hazard for MAKE was predicted by reduced kidney function, and non-commercial health insurance at the time of FSGS identification, as well as prescriptions for non-corticosteroid immunomodulators, hospitalizations, and more frequent outpatient visits. On the other hand, older age was associated with lower hazard for MAKE, implying that younger age might be an important factor in more aggressive forms of FSGS presentation, as was previously demonstrated [23], and that these individuals should likely be considered for early diagnostic biopsy to guide therapeutic intervention.

The subgroup analyses by eGFR category provided important insights. For example, hypertension was common across all levels of kidney function, and patients with reduced kidney function were more likely to have non-commercial insurance and to be prescribed ACE inhibitors/ARBs. Importantly, there were trends for lower survival in groups with lower eGFR, and despite few observed events, findings of risk predictors for MAKE by eGFR category were generally consistent with the main analysis.

In an exploratory analysis of the subset of patients with baseline albuminuria or proteinuria measures, having above-median UACR/UPCR (≥ 1,430 mg/g UACR or ≥ 1.6 g/g UPCR) predicted a more than three-fold greater hazard for MAKE compared to study participants with measures below the median, with non-commercial health insurance, reduced kidney function, prescription for ACE inhibitors/ARBs and non-corticosteroid immunomodulators, and hospitalizations, suggesting these patients are sicker and had consistent risks for MAKE. Interestingly, a cohort of US Veteran patients with FSGS and government-funded health insurance had significant healthcare resource utilization, with ~ 40% having inpatient admission and 33% having emergency department visits within the first year following their FSGS diagnosis [24]. However, patients with access to only non-commercial insurance may face added limitations that could further negatively impact their disease diagnosis and treatment, possibly due to poor access to healthcare services, especially visits to specialists. Our study is the first to show that FSGS patients with non-commercial health insurance also have increased risk for MAKE, implying that these patients may need enhanced care. Notably, the increased risk of MAKE observed with prescriptions for ACE inhibitors/ARBs, which are cornerstone therapies to slow proteinuric CKD progression, may potentially be attributed to association with acute decreases in eGFR among hospitalized patients with multiple co-morbidities, hyperkalemia, medication non-adherence [25–28] or confounding by indication. Interestingly, in the albuminuria or proteinuria subset, health system emerged as a predictor of MAKE with lower risk within Providence versus UCLA Health. However, in the main analysis of the current study, there was no difference in MAKE among patients receiving care at either Providence or UCLA Health, which suggests possible differences in populations or care processes, e.g. albuminuria or proteinuria testing, between systems may exist.

Historically, a number of factors have been recognized as predictors of CKD progression in patients with FSGS, but proteinuria remains a hallmark prognostic indicator of poor kidney survival, with > 50% of patients progressing to kidney failure [4, 11, 29, 30]. In FSGS, nephrotic range proteinuria (> 3.5 g/24 h) typically represents significant podocyte effacement and podocyte loss, particularly if podocyte depletion is greater than 40%, in which case this picture is characteristically associated with reduced kidney function [31]. The results of our study are consistent with the known impact of elevated proteinuria, even at levels lower than nephrotic range as a significant predictor of MAKE. In addition to proteinuria, histopathologic lesions have been implicated as highly predictive of poor clinical outcomes in FSGS [32]. In the Nephrotic Syndrome Study Network, a cohort of 224 participants with FSGS who were biopsied between 2010 and 2017, histologic features that were most predictive of poor clinical outcomes in patients included not only the typical lesions of FSGS, but also lesions of interstitial fibrosis and tubular atrophy, among other characteristics such as adhesion, periglomerular fibrosis, and acute tubular injury [32]. While certain histologic variants have important prognostic value that may distinguish primary from secondary forms of FSGS [33–35], no histopathologic lesion is pathognomonic of primary FSGS [3], and as such other clinical features may be more relevant when assessing and predicting risk for clinical outcomes.

Several studies have tested the association of clinical features with various outcomes in FSGS. Age ≥ 60 years and eGFR < 60 mL/min/1.73 m^2^ have been uncovered as independent risks for reaching a 50% decline in eGFR or kidney failure [36]. Higher baseline serum creatinine has also been found to be a positive predictor of kidney failure even when both clinical and histological parameters are evaluated [37, 38]. Similar to our study, however, body mass index and hypertension have not proven to have prognostic value in FSGS [4, 11, 35].

MAKE is now an accepted composite kidney disease outcome and has been routinely used to facilitate comparisons of patient-centered, primary and secondary outcomes across clinical trials [39–41]. Additionally, predictors of MAKE may permit selection of patients who could most benefit from early kidney biopsy to guide clinical decision making. Indeed, predictive models have successfully incorporated combinations of clinical, biochemical, and pathological features to evaluate the risk for CKD progression, kidney failure, and all-cause mortality in FSGS patients [34, 38, 42–46]. However, the inclusion of other indicators such as medication (e.g., ACE inhibitors/ARBs, non-corticosteroid immunomodulators) and health care utilization (e.g., frequency of outpatient visits and hospitalization) may also be useful in risk stratification for more appropriate and timely management of FSGS. With the advent of new and improved therapies for FSGS, predicting MAKE for risk stratification can help facilitate precision care. As such, the results of our study may have important implication for clinical practice indicating that in addition to known clinical factors, there are non-clinical predictors that should be included in algorithms to identify high-risk patients who may benefit from close monitoring and intervention.

### Strengths and limitations

There are several strengths to the current study. The dataset consists of extensively curated data from two large, geographically distinct health systems consisting of demographics, laboratory measurements, active prescription records, and unique parameters of insurance status and records of inpatient and outpatient visits. Of note, CURE-CKD is one of the largest registry cohorts comprised of over 600 patients with FSGS treated in real-world clinical practice. Conversely, the use of EHR data presents limitations including the use of diagnostic codes to establish diagnosis of FSGS, missing data, analysis of retrospective data, potential miscoding of conditions in the absence of a kidney biopsy diagnosis, inability to verify medication taking, race and ethnicity information that may not correctly reflect self-reported race and ethnicity and recording of health insurance status that may change over time. Importantly, a contemporary diagnosis of FSGS may be designated by clinical parameters without kidney biopsy, therefore some clinical diagnoses of FSGS may not have been confirmed by histopathology. This is considered an important limitation in the current study. As the study was primarily population-based, it was not intended to (re)define the biology of FSGS, as in studies that examined specific histologic predictors in modeling FSGS outcome, or studies that required a biopsy-proven diagnosis to assess response to therapy [47–50], and thus the lack of biopsy data may have impacted the overall study findings. For example, patients with FSGS may have been misclassified, and this may have affected patient selection for immunomodulator therapy. Finally, the study results did not examine post-transplant FSGS, or the effect of responsiveness to treatment and were limited to two, albeit large health systems, and thus may not be generalizable to health systems in different geographic locations.

## Conclusions

In conclusion, this is the largest study to examine unique, population level predictors of MAKE. The study showed that substantial loss of kidney function or kidney failure occurred in more than four in ten patients with FSGS and a mean baseline eGFR of 60 mL/min/1.73 m^2^ by a median of three years. We uncovered both clinical and non-clinical predictors of MAKE in a diverse, adult population from contemporary clinical practices of two large, geographically distinct US health systems. MAKE was predicted by lower baseline kidney function, prescriptions for non-corticosteroid immunomodulators, younger age, and unique features of non-commercial health insurance, and more frequent healthcare utilization in the form of outpatient visits and hospitalization. In essence, the results of our study suggest that these unique population level predictors can facilitate risk stratification to identify patients with FSGS who could most benefit from diagnostic testing and interventions to improve clinical outcomes.

## Figures and Tables

**Table 1:** Characteristics of patients identified with FSGS in 2016–2022

	Total	UACR/UPCR present	UACR/UPCR missing
**Demographics**			
Patients, n (% of total)	629 (100.0)	299 (47.5)	330 (5.2)
Gender, n (%)			
Women	287 (45.6)	134 (44.8)	153 (46.4)
Men	342 (54.4)	165 (55.2)	177 (53.6)
Race and ethnicity, n (%)			
American Indian or Alaska Native	10 (1.6)	3 (1.0)	7 (2.1)
Asian	83 (13.2)	45 (15.1)	38 (11.5)
Black	55 (8.7)	24 (8.0)	31 (9.4)
Hispanic or Latino(a)	28 (4.5)	12 (4.0)	16 (4.8)
Native Hawaiian or Pacific Islander	8 (1.3)	5 (1.7)	3 (0.9)
White	327 (52.0)	142 (47.5)	185 (56.1)
Other^1^ or missing	118 (18.8)	68 (22.7)	50 (15.2)
Age, y, mean, SD	53, 17	53, 18	52, 17
Age Category			
18–39	183 (29.1)	86 (28.8)	97 (29.4)
40–59	206 (32.8)	93 (31.1)	113 (34.2)
60–79	214 (34.0)	106 (35.5)	108 (32.7)
≥80	26 (4.1)	14 (4.7)	12 (3.6)
Primary health insurance, n (%)			
Commercial	371 (59.0)	188 (62.9)	183 (55.5)
Medicaid	75 (11.9)	28 (9.4)	47 (14.2)
Medicare	169 (26.9)	81 (27.1)	88 (26.7)
Missing/Unknown	14 (2.2)	2 (0.7)	12 (3.6)
**Health System Characteristics and Health Care Utilization**			
Health System, n (%)			
UCLA Health	204 (32.4)	111 (37.1)	93 (28.2)
Providence	425 (67.6)	188 (62.9)	237 (71.8)
Outpatient Visits, median (IQR)	18 (8–33)	23 (12–38)	12 (6–29)
Hospitalization, n (%)	181 (28.8)	73 (24.4)	108 (32.7)
**Baseline Medication Coverage (≥45 d), n (%)**			
Medication use, n (%)			
ACE inhibitor/ARB	475 (75.5)	237 (79.3)	238 (72.1)
Corticosteroid	297 (47.2)	139 (46.5)	158 (47.9)
Other immunomodulator^2^	74 (11.8)	29 (9.7)	45 (13.6)
SGLT2 inhibitor	22 (3.5)	15 (5.0)	7 (2.1)
**Baseline Clinical Characteristics**			
Hypertension, n (%)	535 (85.1)	264 (88.3)	271 (82.1)
Diabetes, n (%)	245 (39.0)	140 (46.8)	105 (31.8)
eGFR, mL/min/1.73m^2^, n (%)	629 (100.0)	299 (100.0)	330 (100.0)
mean, SD	60, 30	60, 30	60, 30
eGFR category, n (%)			
≥90	128 (20.3)	60 (20.1)	68 (20.6)
60–89	153 (24.3)	75 (25.1)	78 (23.6)
45–59	104 (16.5)	44 (14.7)	60 (18.2)
30–44	140 (22.3)	69 (23.1)	71 (21.5)
15–29	104 (16.5)	51 (17.1)	53 (16.1)
UACR, mg/g, n (%)	233 (37.0)	233 (77.9)	-
median (IQR)	1430 (520-2630)	1430 (520–2630)	-
UPCR, g/g, n (%)	119 (18.9)	119 (39.8)	-
median (IQR)	1.6 (0.5–3.9)	1.6 (0.5–3.9)	-
Systolic blood pressure, mmHg, n (%)	592 (94.1)	289 (96.7)	303 (91.8)
mean, SD	132, 16	133, 16	132, 17
BMI, n (%)	580 (92.2)	281 (94.0)	299 (90.6)
mean, SD	30, 7	30, 8	30, 7
HbA1c, %, n (% of diabetes)	159 (64.9)	100 (71.4)	59 (56.2)
mean, SD	7.0,1.6	7.1,1.8	6.9, 1.4

FSGS-Focal Segmental Glomerulosclerosis, UACR-urine albumin/creatinine ratio, UPCR-urine protein/creatinine ratio, SD-standard deviation, UCLA-University of California, Los Angeles, IQR-interquartile range, ACE-angiotensin converting enzyme, ARB-angiotensin II receptor blocker, SGLT-sodium-glucose co-transporter, eGFR-estimated glomerular filtration rate; BMI-body mass index, Hb-hemoglobin.

1 includes patients that did not identify with main census categories.

2 includes biologics, calcineurin inhibitors, cytotoxic agents, mTOR inhibitors, and pyrimidine synthesis inhibitors.

**Table 2:** Summary of outcomes among study participants with FSGS and MAKE over median follow-up of 2.9 years (2016–2022)

	Total	Measured for UACR/UPCR	Not Measured for UACR/UPCR
Total with MAKE, n (% of cohort)	262 (41.7)	125 (41.8)	137 (41.5)
**Event type, n (% of N with MAKE)**			
eGFR ≥ 40% decline	133 (50.8)	62 (49.6)	71 (51.8)
Kidney failure or kidney replacement therapy			
eGFR <15 mL/min/1.73 m^2^	74 (28.2)	33 (26.4)	41 (29.9)
Dialysis	28 (10.7)	17 (13.6)	11 (8.0)
Transplant	13 (5.0)	6 (4.8)	7 (5.1)
All-cause death	14 (5.3)	7 (5.6)	7 (5.1)

MAKE-major adverse kidney events, FSGS-focal segmental glomerulosclerosis, UACR-urine albumin/creatinine ratio, UPCR-urine protein/creatinine ratio, eGFR-estimated glomerular filtration rate

## Data Availability

The data that support the findings of this study are available on request from the corresponding author. The data are not publicly available due to privacy or ethical restrictions and would require a data use agreement.

## Abbreviations

FSGS: Focal segmental glomerulosclerosis
US: United States
MAKE: Major adverse kidney events
ACE: Angiotensin converting enzyme
ARB: Angiotensin receptor blockers
eGFR: Estimated glomerular filtration rate
UCLA: University of California, Los Angeles Health
CURE-CKD: Center for Kidney Disease Research, Education, and Hope
EHR: Electronic health records for patients with
CKD: Chronic kidney disease
ICD: International Classification of Diseases
SD: Standard deviation
IQR: Interquartile range
UACR: Urine albumin/creatinine ratio
UPCR: Urine protein/creatinine ratio
CI: Confidence interval
HR: Hazard ratio

## References

[1] Rosenberg AZ, Kopp JB: Focal Segmental Glomerulosclerosis. Clin J Am Soc Nephrol 2017, 12(3):502–517. 28242845 10.2215/CJN.05960616 PMC5338705

[2] Sambharia M, Rastogi P, Thomas CP: Monogenic focal segmental glomerulosclerosis: A conceptual framework for identification and management of a heterogeneous disease. Am J Med Genet C Semin Med Genet 2022, 190(3):377–398. 35894442 10.1002/ajmg.c.31990 PMC9796580

[3] KDIGO 2021 Clinical Practice Guideline for the Management of Glomerular Diseases. Kidney Int 2021, 100(4s):S1–s276. 34556256 10.1016/j.kint.2021.05.021

[4] Rydel JJ, Korbet SM, Borok RZ, Schwartz MM: Focal segmental glomerular sclerosis in adults: presentation, course, and response to treatment. Am J Kidney Dis 1995, 25(4):534–542. 7702047 10.1016/0272-6386(95)90120-5

[5] Hommos MS, De Vriese AS, Alexander MP, Sethi S, Vaughan L, Zand L, Bharucha K, Lepori N, Rule AD, Fervenza FC: The Incidence of Primary vs Secondary Focal Segmental Glomerulosclerosis: A Clinicopathologic Study. Mayo Clin Proc 2017, 92(12):1772–1781. 29110886 10.1016/j.mayocp.2017.09.011 PMC5790554

[6] O’Shaughnessy MM, Hogan SL, Thompson BD, Coppo R, Fogo AB, Jennette JC: Glomerular disease frequencies by race, sex and region: results from the International Kidney Biopsy Survey. Nephrol Dial Transplant 2018, 33(4):661–669. 29106637 10.1093/ndt/gfx189 PMC6659026

[7] Rovin BH, Adler SG, Barratt J, Bridoux F, Burdge KA, Chan TM, Cook HT, Fervenza FC, Gibson KL, Glassock RJ : Executive summary of the KDIGO 2021 Guideline for the Management of Glomerular Diseases. Kidney Int 2021, 100(4):753–779. 34556300 10.1016/j.kint.2021.05.015

[8] Chun MJ, Korbet SM, Schwartz MM, Lewis EJ: Focal segmental glomerulosclerosis in nephrotic adults: presentation, prognosis, and response to therapy of the histologic variants. J Am Soc Nephrol 2004, 15(8):2169–2177. 15284302 10.1097/01.ASN.0000135051.62500.97

[9] Korbet SM, Genchi RM, Borok RZ, Schwartz MM: The racial prevalence of glomerular lesions in nephrotic adults. Am J Kidney Dis 1996, 27(5):647–651. 8629623 10.1016/s0272-6386(96)90098-0

[10] Laurin LP, Gasim AM, Derebail VK, McGregor JG, Kidd JM, Hogan SL, Poulton CJ, Detwiler RK, Jennette JC, Falk RJ : Renal Survival in Patients with Collapsing Compared with Not Otherwise Specified FSGS. Clin J Am Soc Nephrol 2016, 11(10):1752–1759. 27445167 10.2215/CJN.13091215 PMC5053801

[11] Stamellou E, Nadal J, Hendry B, Mercer A, Bechtel-Walz W, Schiffer M, Eckardt KU, Kramann R, Moeller MJ, Floege J: Long-term outcomes of adults with FSGS in the German Chronic Kidney Disease cohort. Clin Kidney J 2024, 17(7):sfae131. 38989280 10.1093/ckj/sfae131 PMC11234294

[12] Wehrmann M, Bohle A, Held H, Schumm G, Kendziorra H, Pressler H: Long-term prognosis of focal sclerosing glomerulonephritis. An analysis of 250 cases with particular regard to tubulointerstitial changes. Clin Nephrol 1990, 33(3):115–122. 2323110

[13] Forster BM, Nee R, Little DJ, Greasley PJ, Hughes JB, Gordon SM, Olson SW: Focal Segmental Glomerulosclerosis, Risk Factors for End Stage Kidney Disease, and Response to Immunosuppression. Kidney360 2021, 2(1):105–113. 35368810 10.34067/KID.0006172020 PMC8785735

[14] Tuttle KR, Alicic RZ, Duru OK, Jones CR, Daratha KB, Nicholas SB, McPherson SM, Neumiller JJ, Bell DS, Mangione CM : Clinical Characteristics of and Risk Factors for Chronic Kidney Disease Among Adults and Children: An Analysis of the CURE-CKD Registry. JAMA Netw Open 2019, 2(12):e1918169. 31860111 10.1001/jamanetworkopen.2019.18169 PMC6991307

[15] Norris KC, Duru OK, Alicic RZ, Daratha KB, Nicholas SB, McPherson SM, Bell DS, Shen JI, Jones CR, Moin T : Rationale and design of a multicenter Chronic Kidney Disease (CKD) and at-risk for CKD electronic health records-based registry: CURE-CKD. BMC Nephrol 2019, 20(1):416. 31747918 10.1186/s12882-019-1558-9 PMC6868861

[16] Inker LA, Eneanya ND, Coresh J, Tighiouart H, Wang D, Sang Y, Crews DC, Doria A, Estrella MM, Froissart M : New Creatinine- and Cystatin C-Based Equations to Estimate GFR without Race. N Engl J Med 2021, 385(19):1737–1749. 34554658 10.1056/NEJMoa2102953 PMC8822996

[17] SSA’s Death Information, https://www.ssa.gov/dataexchange/request_dmf.html

[18] Statewide Death Profiles, https://data.chhs.ca.gov/dataset/statewide-death-profiles

[19] R Core Team: A language and environment for statistical computing. https://www.R-project.org/ Accessed 6/21/2022. In. R Foundation for Statistical Computing.

[20] Therneau T AE, Crowson C: A Package for Survival Analysis in R_. R package version 3.3–1. https://github.com/therneau/survival. Acessed 6/21/2023. . In.; 2023.

[21] TA G: _prodlim: Product-Limit Estimation for Censored Event History Analysis_. R package version 2023.03.31. https://CRAN.R-project.org/package=prodlim. In.; 2013.

[22] What’s the difference between Medicare and Medicaid? Accessed 10/2/2024. https://www.hhs.gov/answers/medicare-and-medicaid/what-is-the-difference-between-medicare-medicaid/index.html

[23] Tuttle KR, Abner CW, Walker PD, Wang K, Rava A, Heo J, Bunke M: Clinical Characteristics and Histopathology in Adults With Focal Segmental Glomerulosclerosis. Kidney Med 2024, 6(2):100748. 38196777 10.1016/j.xkme.2023.100748 PMC10772385

[24] Goldschmidt D, Bensink ME, Zhou ZY, Shi S, Lin Y, Shi L: Epidemiology and burden of focal segmental glomerulosclerosis among United States Veterans: An analysis of Veteran’s Affairs data. PLoS One 2024, 19(12):e0315302. 39671357 10.1371/journal.pone.0315302 PMC11642916

[25] Whiting P, Morden A, Tomlinson LA, Caskey F, Blakeman T, Tomson C, Stone T, Richards A, Savović J, Horwood J : What are the risks and benefits of temporarily discontinuing medications to prevent acute kidney injury? A systematic review and meta-analysis. BMJ Open 2017, 7(4):e012674. 10.1136/bmjopen-2016-012674 PMC5541442 28389482

[26] Mansfield KE, Nitsch D, Smeeth L, Bhaskaran K, Tomlinson LA: Prescription of renin-angiotensin system blockers and risk of acute kidney injury: a population-based cohort study. BMJ Open 2016, 6(12):e012690. 10.1136/bmjopen-2016-012690 PMC5223684 28003286

[27] Tomson C, Tomlinson LA: Stopping RAS Inhibitors to Minimize AKI: More Harm than Good? Clin J Am Soc Nephrol 2019, 14(4):617–619. 30814113 10.2215/CJN.14021118 PMC6450359

[28] Wawruch M, Petrova M, Celovska D, Alfian SD, Tesar T, Murin J, Trnka M, Paduch T, Aarnio E: Non-persistence with multiple secondary prevention medications for peripheral arterial disease among older hypertensive patients. Front Pharmacol 2024, 15:1464689. 39744138 10.3389/fphar.2024.1464689 PMC11688177

[29] Troost JP, Trachtman H, Spino C, Kaskel FJ, Friedman A, Moxey-Mims MM, Fine RN, Gassman JJ, Kopp JB, Walsh L : Proteinuria Reduction and Kidney Survival in Focal Segmental Glomerulosclerosis. Am J Kidney Dis 2021, 77(2):216–225. 32791086 10.1053/j.ajkd.2020.04.014 PMC7854818

[30] Wen Y, Shah S, Campbell KN: Molecular Mechanisms of Proteinuria in Focal Segmental Glomerulosclerosis. Front Med (Lausanne) 2018, 5:98. 29713631 10.3389/fmed.2018.00098 PMC5912003

[31] Wharram BL, Goyal M, Wiggins JE, Sanden SK, Hussain S, Filipiak WE, Saunders TL, Dysko RC, Kohno K, Holzman LB : Podocyte depletion causes glomerulosclerosis: diphtheria toxin-induced podocyte depletion in rats expressing human diphtheria toxin receptor transgene. J Am Soc Nephrol 2005, 16(10):2941–2952. 16107576 10.1681/ASN.2005010055

[32] Zee J, Liu Q, Smith AR, Hodgin JB, Rosenberg A, Gillespie BW, Holzman LB, Barisoni L, Mariani LH: Kidney Biopsy Features Most Predictive of Clinical Outcomes in the Spectrum of Minimal Change Disease and Focal Segmental Glomerulosclerosis. J Am Soc Nephrol 2022, 33(7):1411–1426. 35581011 10.1681/ASN.2021101396 PMC9257823

[33] D’Agati VD, Alster JM, Jennette JC, Thomas DB, Pullman J, Savino DA, Cohen AH, Gipson DS, Gassman JJ, Radeva MK : Association of histologic variants in FSGS clinical trial with presenting features and outcomes. Clin J Am Soc Nephrol 2013, 8(3):399–406. 23220425 10.2215/CJN.06100612 PMC3586971

[34] Shiiki H, Nishino T, Uyama H, Kimura T, Nishimoto K, Iwano M, Kanauchi M, Fujii Y, Dohi K: Clinical and morphological predictors of renal outcome in adult patients with focal and segmental glomerulosclerosis (FSGS). Clin Nephrol 1996, 46(6):362–368. 8982551

[35] Sethi S, Zand L, Nasr SH, Glassock RJ, Fervenza FC: Focal and segmental glomerulosclerosis: clinical and kidney biopsy correlations. Clin Kidney J 2014, 7(6):531–537. 25503953 10.1093/ckj/sfu100 PMC4240407

[36] He HG, Wu CQ, Ye K, Zeng C, Huang YY, Luo SW, Yin W, Ye QR, Peng XM: Focal segmental glomerulosclerosis, excluding atypical lesion, is a predictor of renal outcome in patients with membranous nephropathy: a retrospective analysis of 716 cases. BMC Nephrol 2019, 20(1):328. 31438882 10.1186/s12882-019-1498-4 PMC6704573

[37] Korbet SM: Clinical picture and outcome of primary focal segmental glomerulosclerosis. Nephrol Dial Transplant 1999, 14 Suppl 3:68–73. 10.1093/ndt/14.suppl_3.68 10382985

[38] Greenwood AM, Gunnarsson R, Neuen BL, Oliver K, Green SJ, Baer RA: Clinical presentation, treatment and outcome of focal segmental glomerulosclerosis in Far North Queensland Australian adults. Nephrology (Carlton) 2017, 22(7):520–530. 27170059 10.1111/nep.12816

[39] Flannery AH, Bosler K, Ortiz-Soriano VM, Gianella F, Prado V, Lambert J, Toto RD, Moe OW, Neyra JA: Kidney Biomarkers and Major Adverse Kidney Events in Critically III Patients. Kidney360 2021, 2(1):26–32. 35368827 10.34067/KID.0003552020 PMC8785730

[40] Semler MW, Rice TW, Shaw AD, Siew ED, Self WH, Kumar AB, Byrne DW, Ehrenfeld JM, Wanderer JP: Identification of Major Adverse Kidney Events Within the Electronic Health Record. J Med Syst 2016, 40(7):167. 27234478 10.1007/s10916-016-0528-z PMC5791539

[41] Wheeler DC, Stefánsson BV, Jongs N, Chertow GM, Greene T, Hou FF, McMurray JJV, Correa-Rotter R, Rossing P, Toto RD : Effects of dapagliflozin on major adverse kidney and cardiovascular events in patients with diabetic and non-diabetic chronic kidney disease: a prespecified analysis from the DAPA-CKD trial. Lancet Diabetes Endocrinol 2021, 9(1):22–31. 33338413 10.1016/S2213-8587(20)30369-7

[42] Cai Y, Liu Y, Tong J, Jin Y, Liu J, Hao X, Ji Y, Ma J, Pan X, Chen N : Develop and Validate a Risk Score in Predicting Renal Failure in Focal Segmental Glomerulosclerosis. Kidney Dis (Basel) 2023, 9(4):285–297. 37899999 10.1159/000529773 PMC10601954

[43] Chitalia VC, Wells JE, Robson RA, Searle M, Lynn KL: Predicting renal survival in primary focal glomerulosclerosis from the time of presentation. Kidney Int 1999, 56(6):2236–2242. 10594800 10.1038/sj.ki.4491164

[44] Ossareh S, Yahyaei M, Asgari M, Bagherzadegan H, Afghahi H: Kidney Outcome in Primary Focal Segmental Glomerulosclerosis (FSGS) by Using a Predictive Model. Iran J Kidney Dis 2021, 15(6):408–418. 10.52547/ijkd.6442 34930852

[45] Weng Q, Zhou Q, Tong J, Jin Y, Liu Y, Yu X, Pan X, Ren H, Wang W, Xie J : New risk score for predicting steroid resistance in patients with focal segmental glomerulosclerosis or minimal change disease. Clin Proteomics 2020, 17:18. 32514258 10.1186/s12014-020-09282-x PMC7257237

[46] Zhu Y, Xu W, Wan C, Chen Y, Zhang C: Prediction model for the risk of ESKD in patients with primary FSGS. Int Urol Nephrol 2022, 54(12):3211–3219. 35776256 10.1007/s11255-022-03254-w

[47] Trachtman H, Nelson P, Adler S, Campbell KN, Chaudhuri A, Derebail VK, Gambaro G, Gesualdo L, Gipson DS, Hogan J : DUET: A Phase 2 Study Evaluating the Efficacy and Safety of Sparsentan in Patients with FSGS. J Am Soc Nephrol 2018, 29(11):2745–2754. 30361325 10.1681/ASN.2018010091 PMC6218860

[48] Shoji J, Goggins WC, Wellen JR, Cunningham PN, Johnston O, Chang SS, Solez K, Santos V, Larson TJ, Takeuchi M : Efficacy and Safety of Bleselumab in Preventing the Recurrence of Primary Focal Segmental Glomerulosclerosis in Kidney Transplant Recipients: A Phase 2a, Randomized, Multicenter Study. Transplantation 2024, 108(8):1782–1792. 39042770 10.1097/TP.0000000000004985 PMC11262731

[49] Thurman JM, Wong M, Renner B, Frazer-Abel A, Giclas PC, Joy MS, Jalal D, Radeva MK, Gassman J, Gipson DS : Complement Activation in Patients with Focal Segmental Glomerulosclerosis. PLoS One 2015, 10(9):e0136558. 26335102 10.1371/journal.pone.0136558 PMC4559462

[50] Wei C, Trachtman H, Li J, Dong C, Friedman AL, Gassman JJ, McMahan JL, Radeva M, Heil KM, Trautmann A : Circulating suPAR in two cohorts of primary FSGS. J Am Soc Nephrol 2012, 23(12):2051–2059. 23138488 10.1681/ASN.2012030302 PMC3507361

